# Somatic embryogenesis in mature zygotic embryos of *Picea pungens*

**DOI:** 10.1038/s41598-021-98511-w

**Published:** 2021-09-24

**Authors:** Jing Tao, Shigang Chen, Caiyun Qin, Qingmei Li, Jufeng Cai, Changbin Sun, Weiming Wang, Yuhui Weng

**Affiliations:** 1 Jilin Provincial Academy of Forestry Sciences, 3528 Linhe St., Changchun, Jilin China; 2 Changchun Academy of Forestry, 5840 Jingyue St., Changchun, Jilin China; 3 AbrorGen Inc., 2011 Broadbank Court, Ridgeville, SC 29472 USA; 4 Arthur Temple College of Forestry and Agriculture, Stephen F. Austin State University, Nacogdoches, TX 75965 USA

**Keywords:** Biotechnology, Plant sciences

## Abstract

This study developed somatic embryogenesis protocols for *Picea pungens* (Engelm), an important ornamental species, including initiation, proliferation, maturation, germination, and acclimation. Somatic embryogenic tissues were induced from mature zygotic embryos of five families, with a frequency of $\ge$ 22% for each. Embryogenic tissues (ET) from 13 clones of three families were proliferated for one week, achieving an average rate of 179.1%. The ET of 38 clones of three families were cultured in maturation medium for six weeks; 188 mature embryos on average were counted per gram ET cultured, of which $\ge$ 81.1% appeared normal, and each clone developed at least 28 normally matured embryos. A total of 69.9% or more of cotyledonary somatic embryos germinated normally and developed into normal emblings. The experiment of transplanting the emblings into a greenhouse had an average survival rate of 68.5%. Considerable variation among and within families during initiation and proliferation was observed, but this variation decreased in the maturation and germination. Changing the concentration of plant growth regulator of the initiation medium did not significantly change the initiation frequency. We recommend incorporating these protocols into the current *Picea pungens* practical programs, although further research is essential to increase efficiencies and reduce cost.

## Introduction

Advantages of incorporating somatic embryogenesis (SE) into a forest genetic selection and deployment program have been well reported^1–3^. Successful examples in this regard can be found in many commercial species, particularly spruce, such as *Picea abies*^4^, *Picea sitchensis*^5^, *Picea glauca*^6–8^, and *Picea mariana*^6,9^. The operational practice of SE techniques has been incorporated into the commercial forestry of *Picea glauca* and *Picea mariana* since 1990^9,10^.

Valued mainly for its appearance due to its unique, year-round silvery blue-green needle color, *Picea pungens* (Engelm.) has been planted extensively as an ornamental species in North America and Europe^11^. *Picea pungens* has been used in northeast China as an important ornamental species since 2000^12,13^. Demand for planting the species has been increasing in the past 20 years, and consequently, seed production areas have been established^12^. Planting *Picea pungens* using seeds, however, is associated with issues, including difficulty in retaining desirable parental tree characteristics, in particular needle color. Thus, propagation methods that retain ideal characteristics of selected genotypes and allow for efficient regeneration of plants on a large scale are ideal. Artificial vegetative propagation using cuttings or grafting produces the same genotype (clone) as the donor tree, and the effectiveness of these methods is often dependent on the age and physiological state of the donor plant, limiting its practical application in forestry. Given the success of SE in spruce^9,10^, vegetative propagation of *Picea pungens* via the SE method is becoming attractive since the method can overcome the problems associated with cuttings or grafting. An embryogenic cell line established from one seed can generate a high number of somatic embryos; therefore, SE can produce a large number of genetically identical plants within a short period. Nonetheless, SE is a complicated process of multiple steps, including initiation, proliferation, maturation, germination, and acclimation, and for a successful SE method, a satisfactory result is indispensable for each step.

Despite the great desire to apply SE techniques to produce *Picea pungens* seedlings, relevant studies have been limited in the literature. For this species, successful SE initiation and maturation were first reported by Afele et al.^14^, although their efficiencies were relatively low. In subsequent studies^15,16^, great improvement in efficiency was made in initiation and proliferation, but satisfactory results were not achieved in maturation or germination. Clearly, obstacles to SE embryo maturation and germination remain. Furthermore, to our knowledge, data for the subsequent step, acclimation, have been lacking, and such data are essential to assess the feasibility of using SE techniques in deployment. Overall, the techniques achieved thus far have not been sufficiently refined, particularly for maturation and the subsequent steps, limiting the application of SE to *Picea pungens*.

While modification and refinement of protocols continue to be an important aspect of SE research, other topics, such as genetic influence, can play an important role during the SE process. This has been confirmed in some spruce species^5,9,17^ but is not well known for *Picea pungens,* since all previous studies used a single genotype^13–15^. The genetic effect on SE is complicated, depending on the SE stages^16^. An effective SE program for economically important tree species needs protocols that can be applied to a large number of genotypes.

A project to develop protocols for applying SE in *Picea pungens* breeding and deployment was initiated in late 2000 at the Jilin Academy of Forest Science and Research in NE China. Since SE protocols for *Picea glauca* and *Picea mariana* have been well refined and successfully incorporated into commercial seedling propagation in New Brunswick, Canada^9^, in the present study, we adopted those protocols as the foundation, with some adjustments, to *Picea pungens*. By using mature zygotes of *Picea pungens* as materials, all steps of the SE process, starting from embryo initiation to the transfer of emblings to the greenhouse, were evaluated. Thus, the objectives of the present study were (1) to develop protocols for the SE of *Picea pungens* and (2) to investigate whether SE efficiencies vary with family and clone. Initiation is key to spruce SE and may vary with changes in plant growth regulator (PGR) concentrations; thus, a third objective was to test the feasibility of improving initiation frequency by changing PGR concentrations.

## Materials and experiments

In 2008, stored mature *Picea pungens* seeds from five families, representing diverse origins from New Brunswick (Family 113 and 8285450, gifted from New Brunswick Department of Natural Resources and National Tree Seed Center, Natural Resources Canada, respectively), Nova Scotia (200301, purchased from Nova Tree Seed Company, Inc., Truro, Nova Scotia), and Ontario (OSC, purchased from Ontario Seed Center, Waterloo, Ontario) in Canada and New York, USA (10910, purchased from TreeHelp.com, Buffalo, New York), were used as materials. Prior to the experiments, all seeds were stored at low temperature for two or more years.

Prior to embryo extraction, all seeds were submerged in 75% ethanol for 1 min, rinsed three times in sterile water, soaked in 10% sodium hypochlorite with a small amount of Tween-20 for 30 min, rinsed five times in sterile water, and then kept in sterile water for 4 h to soften the seed coat. Mature zygotic embryos were extracted from seeds under a stereomicroscope. Experiments for key SE steps were then carried out, and details for the respective medium supplements and PGR concentrations are presented in Table 1 and for the experimental materials in Table 2.

**Table 1:** Plant growth regulator (PGR) treatments and concentrations and supplement contents were applied by somatic embryogenesis. Note that the base medium was half-strength Litvay’s medium.

Step	PGR concentration (μM)	Supplement (g/L)
Initiation	Low: BA 1.25 + 2,4-D 2.5 BA 2.5 + 2,4-D 5.0 Standard: BA 5.0 + 2,4-D 10.0 High: BA 7.5 + 2,4-D 12.5 BA 7.5 + 2,4-D 15.0 BA 10.0 + 2,4-D 15.0 BA 10.0 + 2,4-D 20.0	Sucrose 10.0 Gelrite 4.0 Glutamine 0.5
Proliferation	Liquid: BA 3.75 + 2,4-D 6.25	Sucrose 10.0 Glutamine 0.5
	Solid: BA 7.5 + 2,4-D 12.5	Sucrose 10.0 Gelrite 4.0 Glutamine 0.5
Maturation	ABA 60.0	Sucrose 60.0 Gelrite 6.0 Glutamine 0.5
Germination^a^		Sucrose 20.0 Gelrite 6.0 Glutamine 0.5

^a^Contents for all half-strength Litvay’s medium elements were reduced to half other than KNO_3_, KH_2_PO_4_, and Fe-EDTA.

**Table 2:** Summary of the number of families, the number of clones per family, the total cultured tissues, the total successful tissues and the success rate by somatic embryogenesis step.

Step	Number of families		Number of clones per family	Total cultured^a^	Total success^a^	Rate
Initiation	5		79	2902	979	0.34
Proliferation	3		13	4.3	11.9	2.79
Maturation	3		38	8324	6751	0.81
Germination	2		13	633	443	0.70
Acclimation	2		7	261	179	0.69

^a^Units for total cultured and total success were the total number of zygotic embryos cultured and the number of embryos with established embryogenic tissues for initiation, weight (g) of embryogenic tissues before and after the liquid culture period for proliferation, the number of total matured and the number of normally matured embryos for maturation, the number of total matured embryos planted and the number of emblings developed for germination, the number of emblings planted, and the number of emblings that successfully developed for acclimation, respectively.

### Exp. 1: Initiation of embryogenic tissue

The initiation medium was half-strength Litvay’s medium^18^ (½ LM) supplemented with sucrose, l-glutamine, gelrite, and a (standard) PGR concentration combination containing 10.0 μM 2,4-dichlorophenoxyacetic acid (2,4-D) and 5.0 μM benzyl aminopurine (BA) (referred to as the standard PGR concentration; Table 1). To investigate the effects of PGR concentration, two PGR concentration treatments, low and high, were also tested. The low treatment included two concentration combinations containing $\le$ 5.0 μM 2,4-D and $\le$ 2.5 μM BA, and the high treatment had four concentration combinations containing $\ge$ 12.5 μM 2,4-D and $\ge$ 7.5 μM BA (Table 1). The pH of the medium was adjusted to 5.8 prior to sterilization.

All five families, with an average of 79 (range 75–94) mature zygotic embryos per family, were cultured on the initiation media in 100 × 15-mm Petri dishes containing approximately 25 ml of medium (approximately 10–15 embryos per dish and approximately 10 dishes per family × PGR combination) in darkness at 24 $\pm$ 1 °C. One month later, SE initiation was assessed by recording the success or failure of each cultured embryo. Success was defined for initiation of an embryo if embryogenic tissue (ET) was established. The initiation frequency (= number of embryos with successful initiation/number of cultured embryos) was then calculated to indicate initiation efficiency. Once an embryo had developed ET, it was given a clone identification number. Note that a clone (embryogenic line) in this study refers to embryogenic tissues initiated from a single embryo.

### Exp. 2: Proliferation of embryogenic tissue

Once an ET clone was identified, a rapid proliferation of ET for cryopreservation and/or production was needed. This experiment used ET from 12 clones representing three families (113, 8285450 and OCS) obtained in Exp. 1. ET was proliferated via a method of two steps: liquid suspension culture on liquid proliferation medium, followed by culture on solid proliferation medium. The base proliferation medium was the same as the initiation medium, but the PGR combination varied, being 3.75 µM BA and 6.25 µM 2,4-D for liquid and 7.5 µM BA and 12.5 µM 2,4-D for solid cultures (Table 1).

Before transferring to the proliferation medium, ET was cultured on medium without BA and 2,4-D for seven days. Approximately 30–40 mg ET per clone was subcultured in flasks (10–15 mg ET per flask) with 50 ml liquid proliferation medium for seven days. During this period, the flasks were shaken vigorously (121 RPM) to break up the tissue pieces into a fine suspension. The suspension in a flask was poured onto filter paper to remove liquid, and the filter paper with attached cells was placed onto the surface of fresh, solid proliferation medium. The tissues were cultured in darkness at approximately 24°$\pm$ 1 °C for two weeks and then subcultured every 14 days on the solid medium. ET weights for each flash were recorded before and after the liquid culture period, and the percentage increase (= $\frac{after-before}{before}\times 100$) was used as the response variable for proliferation efficiency. Due to its difficult measurement, proliferation in solid culture was not recorded.

### Exp. 3: Maturation of somatic embryos

This experiment was conducted using ET from 38 clones initiated from three families (113, 8285450 and OCS) in Exp. 1 and 2. The maturation medium was 1/2 LM containing 6% sucrose, 0.6% gelrite, 0.05% glutamine, and 60 $\upmu$M abscisic acid (ABA) (Table 1).

Before transferring to the maturation medium, ET was subcultured on solid proliferation medium for two periods, each period being 7 days, and then cultured on proliferation medium without BA and 2,4-D for another seven days. ET clumps that grew vigorously were selected for maturation. 3–8 Petri dishes each with 6–8 clumps (approximately 0.5 g) were prepared for each clone. Cultures were kept at approximately 24 °C, low light intensity (5 mol/$\upmu$m^2^/s, cool white, fluorescent lamps GE F72T12/CW), and a 16-h photoperiod for six weeks without subculturing onto fresh medium. By the end of the culture period, the total number of normal embryos (1.5–2 mm in length, having 2–5 distinct cotyledons, embryonal root caps, and smooth hypocotyls) and abnormal embryos were counted and expanded on a per gram basis. The number of normally matured embryos per gram was used as the indicator of maturation efficiency.

### Exp. 4: Germination and rooting

This experiment involved 13 clones representing two different families (8285450 and OCS) from Exp. 3. The germination medium was a modified ½ LM paired with 2% sugar, 0.6% Gelrite, 0.05% l-Glutamine and no PGRs (Table 1).

Matured embryos at the cotyledonary stage in Exp. 3 were isolated and placed onto germination medium (20 mature embryos per dish and approximately three dishes per clone) for culture under similar environmental conditions as for maturation. After 1 week of culture in darkness at 24 $\pm$ 1 °C, cotyledonary and small radicles appeared. After culture for the next four weeks in the light, normal emblings (plantlets germinated from somatic embryos with young hypocotyls of 1–2 cm and radicles of 2–3 cm) had developed. The total number of embryos planted and the number of emblings developed after the culture were recorded. The frequency (= number of normal emblings/number of embryos planted) was calculated to indicate germination efficiency.

### Exp. 5: Acclimation

This experiment used six clones representing one family (8285450) and one clone from family OCS in Exp. 4. On average, 44 emblings per clone with properly developed radicles and hypocotyls were directly transferred into pots (filled with Jiffy peat pellets). For the first two weeks in the greenhouse, the emblings were kept under a 16-h photoperiod at 25 $\pm$ 5 °C and each embling plantlet was kept under a glass beaker to protect against dehydration. The emblings were grown until they reached a height of 10–15 cm (20–25 weeks). The number of emblings that successfully developed was counted, and the survival rate (= number of successfully developed emblings/total number of planted emblings) was calculated as the indicator of acclimation efficiency.

### Data analysis

In Exp 1, the response variable for initiation, the number of embryos with successful initiation out of the total number of embryos cultured (data in ‘event/trial’ type), was assumed to follow a binomial distribution and analyzed using the following models:

1 $$ y_{ijk} = \mu + F_{i} + T_{j} + FT_{ij} + P_{k\left( j \right)} + \varepsilon_{ijk} , $$

where ${y}_{ijk}$ was the event/trial in initiation of the kth petri dish of the ith family and jth PGR treatment and kth PGR concentration combination within the jth PGR treatment. $\mu$ was the overall mean, ${F}_{i}$, ${T}_{i}$, ${P}_{k\left(j\right)}$, and ${FT}_{ij}$ were the fixed effects of the family, PGA treatment, PGR combination within a treatment, and the interaction between *F* and *T*, respectively, and ${\varepsilon }_{ijkl}$ was the random error.

The response variable of Exp 2, the percent ET increase of the kth dish of the jth clone within the ith family (${y}_{ijk}$) is continuous data and, was, therefore, analyzed using analysis of variance following the model below:

2 $$ y_{ijk} = \mu + F_{i} + C_{j\left( i \right)} + \varepsilon_{ijk} , $$

where ${C}_{j\left(i\right)}$ was the fixed effect of the jth clone within the ith family, and the others remained the same as explained previously.

In Exp. 3, the total number of normally matured embryos per gram ET cultured is count data and can be modeled using a Poisson distribution. The data were analyzed using Model [2] but paired with a negative binomial distribution, which is similar to the Poisson distribution but can handle the over-dispersion issue.

In Exp. 4 and 5, the response variable was the ‘event/trial’ type. The numbers of emblings over the embryos cultured on germination medium and the normal emblings developed over the total emblings transferred in acclimation were analyzed using Model [2], where all terms remained the same as previously described.

SAS programs^19^ were used in the analyses (i.e., logistic procedure for the ‘event/trial’ data, GLM procedure for the continuous data, and GENMOD procedure for the count data). Means and their standard errors (SE) were computed either on an original scale directly or transformed to the original scale via a link, depending on the data type. Multiple comparisons among levels of a factor were made using the Tukey method. Pearson correlations at the clonal level among efficiencies of proliferation, maturation, germination, and acclimation were tested for significance. Unless otherwise stated, the significance level in this paper refers to $\alpha$=0.05.

### Research involving plants

The authors declare that this study on plants in this study, including the collection of plant material, complies with relevant institutional, national, and international guidelines and legislation.

## Results

**Figure 1:**
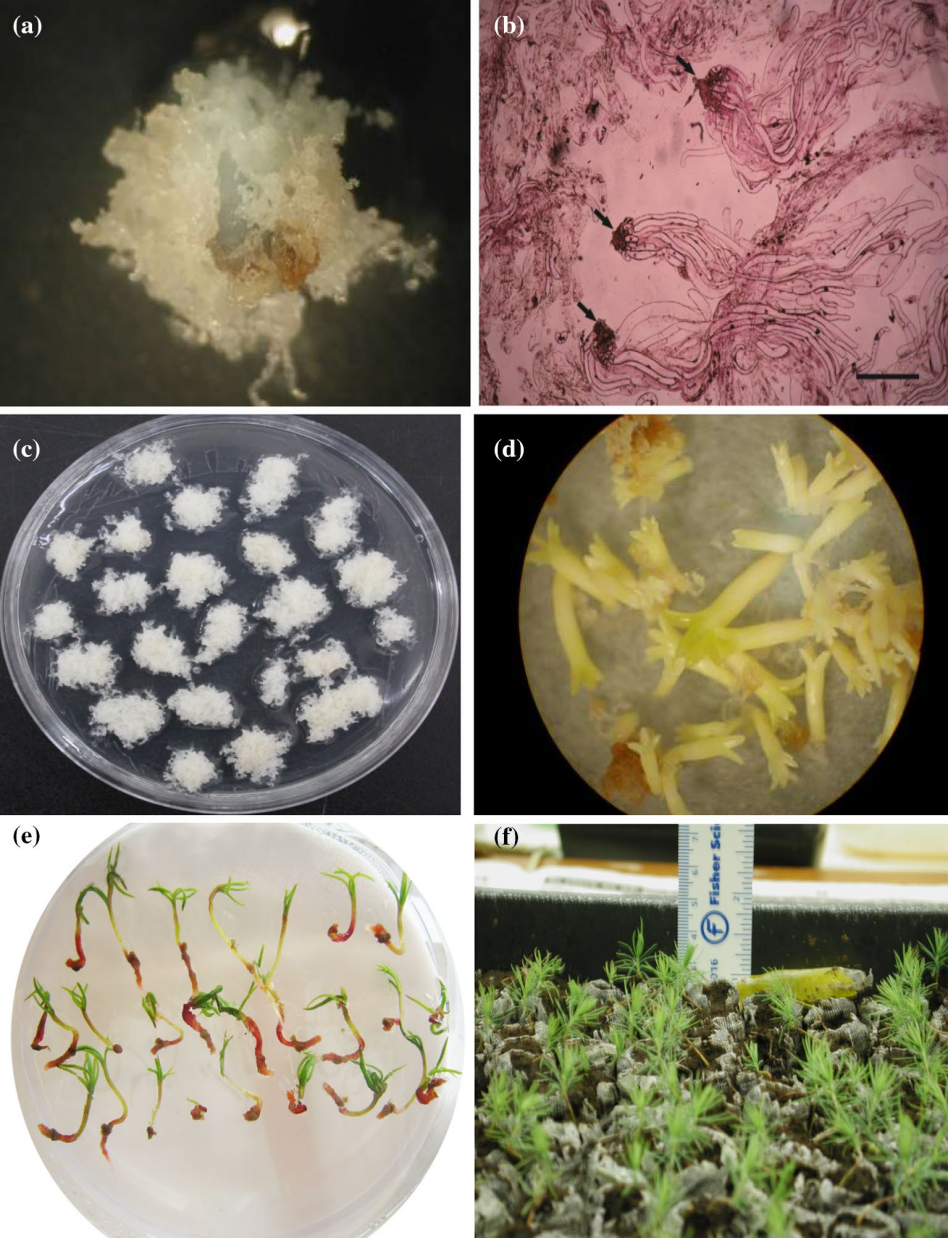
Somatic embryogenesis in *Picea pungens*: (**a**) initiation of embryogenic cell mass; (**b**) embryogenic tissues under a microscope after 14 days proliferation culture (bar = 50 µm); (**c**) embryogenic tissues on solid proliferation medium; (**d**) normally matured somatic embryos under mature conditions; (**e**) embryo germination and rooting; and (**f**) growing embryo seedlings for family 8285450.

**Figure 2:**
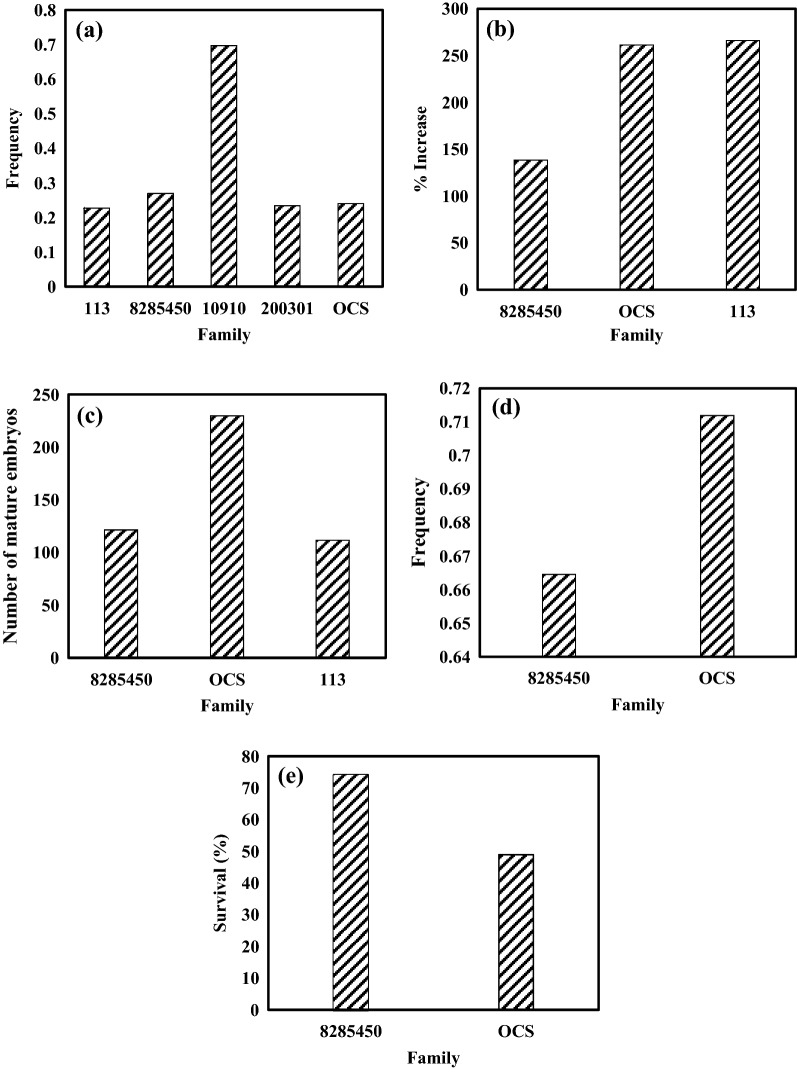
Average and standard error by family: (**a**) initiation frequency; (**b**) proliferation rate (% increase); (**c**) the number of mature embryos per gram embryo tissue; (**d**) germination frequency; and (**e**) embling survival rate in acclimation.

Two weeks post-inoculation, light brown tissues appeared on the zygotic embryo explants, and after another 1–2 weeks, embryos started to produce translucent early embryogenic tissue (Fig. 1a). While all families responded to the initiation media, the frequency differed among families (Table 3; Fig. 2a). The frequencies were comparable (ranging from 0.23 to 0.27) for all families other than family 10910, which had a significantly higher frequency of 0.68, resulting in an overall initiation frequency of 0.34 (Table 2). The impact of PGR treatment on ET induction (Table 3) was insignificant, as was that among the combinations within a treatment (Table 1). Nevertheless, the highest frequency was observed under the high PGR treatment (0.34), which was closely followed by the standard (0.32) and low (0.31) treatments. Family responded similarly to PGR treatments, as demonstrated by their insignificant interaction (Table 3).

**Table 3:** Results of analysis of variance on embryo initiation (Exp. 1).

Source	DF	Chi-Square	Pr > ChiSq
Family	4	287.48	< 0.0001
Treatment	2	3.23	0.1989
Combination (treatment)	4	0.28	0.9910
Family × treatment	8	8.62	0.3750

The initiated ET continued proliferating in the proliferation medium (Fig. 1b). By the end of the liquid culture period, all clones increased their ET fresh weight (Fig. 1c), on average by 179.1% (Table 2). The rate differed significantly by family (Table 4), ranging from 138.3 to 266.0% (Fig. 2b). The rate also varied greatly within families (Table 4); for example, among the eight clones representing the family 8285450, the proliferation rate ranged from 8.7 to 192.5% (Table 5).

**Table 4:** Results of analysis of variance on embryo tissue proliferation, maturation, germination and acclimation (Exp. 2–5).

Source of variation	Proliferation			Maturation			Germination			Acclimation			
	df^a^	F value	Pr	df	Chi-Square	Pr	df	Chi-Square	Pr	df	Chi-Square	Pr	
Family	2	4.34	0.027	2	5.80	0.055	1	0.09	0.769	1	10.36	0.001	
Clone (family)	9	2.66	0.033	24	45.59	0.005	9	6.89	0.649	4	14.96	0.005	

^a^The degree of freedom (df) for the denominator was 20.

**Table 5:** Clonal range of embryo tissue proliferation, maturation, germination and acclimation, sorted by family.

Phase	Family		
	8285450	OCS	113
Proliferation (%)	8.68–192.50	214.29–308.33	176.00–366.67
Maturation (number)	27.78–976.82	144.00–699.97	50.94–475.00
Germination	0.37–0.83	0.27–0.83	0.59–0.79
Acclimation (%)	56.67–87.50		48.94^a^

^a^Only one clone was tested.

Within one month after transfer, the ET started to develop mature somatic embryos, and after two months, the mature embryos were fully developed (Fig. 1d). Many mature embryos were identified, with an average of 188 embryos per gram ET, and the majority (81.1%) of the embryos appeared normal (Table 2). While normally matured embryos were observed in all families and clones (Fig. 2c; Table 5), the differences in number were barely significant between families, but were significant within families (Table 4). For family 8285450, the average number of normally matured embryos by clone varied from 28 to 977 (Table 5). Even at the family level, their practical differences were large; the family OCS developed 230 mature embryos per gram ET, which was more than doubled that of the family 113 (Fig. 2c).

On the germination medium, the somatic embryos elongated their radicle and hypocotyl quickly, followed by the growth of a shoot and a root (Fig. 1e). All families and clones responded to the process; after four weeks of culture, the average frequency of developing emblings was 0.70 (Table 2). Even though the frequencies were statistically comparable between and within families (Table 4), the actual differences were substantial [i.e., for family 8285450, the clonal frequency varied from 0.37 to 0.83 (Table 5)].

In Exp 5, for acclimation, the emblings developed in the germination culture were directly transferred to pots (filled with Jiffy peat pellets) in a greenhouse environment (Fig. 1f). On average, 68.5% of the transferred emblings survived (Table 2). The survival rate varied with family and clones within a family (Table 3). Family 8285450 (74.5%) had a significantly higher survival than OCS (50.4%) (Fig. 2e). At the clonal level, the average survival rate of the family 8285450 ranged from 56.7 to 87.5%.

No particular patterns stood out in efficiency among proliferation, maturation, germination, or acclimation at the family level (Fig. 2). At the clone level, no calculated correlation coefficient values significantly differed from zero. However, proliferation correlated negatively with germination (r = − 0.44) and acclimation (r = − 0.55) to some extent (Table 6).

**Table 6:** Pearson correlation coefficients among clonal averages in proliferation, maturation, germination, and acclimation. Note that none of the coefficients significantly differed from zero at $\alpha$ = 0.05.

Step	Maturation	Germination	Acclimation
Proliferation	− 0.06	− 0.44	− 0.55
Maturation		− 0.10	− 0.11
Germination			− 0.04

## Discussion

In this study, efforts were made to develop practical SE protocols for *Picea pungens*. We used the protocols for *Picea glauca* and *Picea mariana*^8,9^ as the base and made some modifications based on preliminary trials (data not shown) in order to improve efficiency. Important modifications included: (1) a two-stage proliferation process, first in a liquid medium and then on a solid medium, which is different from that of Adams et al.^9^, in which a one-stage, solid medium proliferation was used. Liquid suspension cultures of conifers were preferred for proliferation since they can multiply at higher rates than solid medium culture^20^. In this study the liquid proliferation culture for seven days increased ET by 179% (Fig. 2); and (2) the ABA concentration of maturation medium was increased to 60 μM (Table 1), whereas that of Adams et al.^9^ used 8 μM, a sevenfold increase. A wide range (0.1–25 mg/L) of ABA has been used for the development and maturation of conifer somatic embryos^21^, but the suitable level varied with species and genotype. For *Picea pungens*, the optimal ABA requirement for maturation varied; Afele et al.^14^ found that it depended on PGR treatment in the initiation medium (i.e., 7.6 μM ABA if the initiation medium containing 10 μM NAA or 2,4-D and 5 μM BA) but Sun and Jia^16^ recommended a much higher concentration (2 mg/L). Overall, results following all the steps in this study are promising having achieved efficiencies to levels similar to those for *Picea glauca*^17^ and *Picea mariana*^9,10,17^, one of the most successful spruce SE commercial programs. In supporting our results, the protocols for *Picea glauca*^8^ have also been successfully applied to SE of *Picea sitchensis*^5^.

At the species level, this study represents great improvements in SE efficiencies and techniques relative to previous SE studies on *Picea pungens*. For this species, the first paper^14^ reported an initiation frequency of 0.13, which was then improved upon to 0.21–0.45 in subsequent studies^15,16^. Similarly, great progress has been made in proliferation, with the proliferation rate improving from approximately 15^15^ to 130%^16^. However, in the subsequent steps of maturation and germination, the majority of the embryos appeared abnormal and failed to develop roots^16^. These studies compared efficiencies among various basal media or PGR concentrations and their results varied. For example, DCR medium was the best for the initiation in one study^16^, while others supported the use of ½ LM medium^14,15^. Compared with previous studies^14–16^, the present study was not found to be inferior in terms of initiation or proliferation, and also showed substantial improvements in maturation and germination. Additionally, the present study directly transplanted the emblings from the Petri dishes into containers in a greenhouse environment and is the first demonstration of successful acclimation with satisfactory survival rates. The survival rates, 74% for family 8285450 (Fig. 2) and 87.5% for its best clone (Table 5), were comparable to those reported for *Picea sitchensis* (67%)^5^*, Picea abies* (77%)^22^, *Picea glauca* and *Picea mariana* (above 80%)^10^. Since physicochemical cultural conditions were not always the same among the present and previous studies, it is difficult to attribute the superiority of this study to any particular factors. One thing, which is crucial but often ignored in developing protocols for a SE system, is that physicochemical conditions of one step may affect results of its subsequent steps. While high auxin/cytokinin combinations facilitated SE initiation^16^, there might have a negative effect on the subsequent cultures, in particular on the ability of the somatic embryos to differentiate into plantlets^14^. Also, high ABA concentrations during maturation can inhibit germination. This might partly contribute to the failure in germination in Ref.^16^, where a high ABA amount (2.0 mg/L) was used in maturation. The current study evaluated the SE system involving all key steps and the promising results for each step suggest the practical significance of the protocols, although there may still be room for improvement.

Plant growth regulator is essential for SE initiation. A few studies have investigated the effects of various PGR concentrations on initiation of *Picea pungens*, but their results varied, with some^14,15^ showing insignificant effects and others^16^ a reversal conclusion. The current study supports^14,15^, with no significant difference being found among and within PGR treatments (Table 3), although a trend of slightly increasing initiation frequency with increasing PGR concentration was also found. Recommended PGR concentration combinations for *Picea pungens* initiation varied: One recommended a PGR concentration combination of 5.0 mg/L 2, 4-D, 4.0 mg/L BA, and 2.0 mg/L KT^16^, a concentration combination similar to our high treatment PGR combination (Table 1), whereas others^14,15^ suggested a PGR concentration combination of 9 µM 2,4-D or NAA and 4.5 µM BA, which is similar to our standard treatment. It appears that a wide range of PGR concentration combinations could be effective for *Picea pungens*, although higher concentrations might be preferred for initiation based on our results. However, high PGR concentrations might increase browning and aging of cultured tissues^15^. The impact of PGR on initiation may be species specific; for example, in *Pinus strobus*, the initiation frequency increases from an average of 0.20–0.53 by reducing the 2,4-D and BA concentrations from 9.5 to 2.2 µM and from 4.5 to 2.2 µM, respectively^23^.

The capacity of SE is often genetically determined, and a standard protocol for each SE step may not be equally effective for all genotypes^9,17,24–26^. While all tested families and clones were responsive in each step in the current study, the variation among and within families was substantial, suggesting that, while the wide application of the protocols is plausible, low efficiencies may occur for some genotypes. Note that SE differences among genotypes in the current study might be underestimated due to the limited numbers of families and clones being tested. Clearly, the effect of genotype on the success of SE in the species should be emphasized, and more studies involving more genotypes are required before a definitive conclusion can be derived. The variation varied by SE step: It was significant in initiation and proliferation and became less significant or insignificant in the subsequent steps of maturation and germination (Table 4). Thus, to improve SE efficiency, genetic selection at the initiation or proliferation steps would be more effective. This pattern is in line with the discovery in *Picea glauca* that genetic control was strong in the initiation phase, but its influence steadily declined through the proliferation, maturation, and germination phases^17^. Even for maturation or germination, where variation among and within families was statistically insignificant (Table 4), the biological significance of the variation might be greater than was statistically evident. While a protocol applicable to a broad range of genotypes is indispensable for commercial timber species, a practical program with species of high ornamental value often works with a limited number of genotypes of great commercial value. The significant effect of genetic background reveals that future focus should be on developing more specific media for important seed families or clones of *Picea pungens*.

A satisfactory efficiency for each step is essential for a practical SE program and, thus, negative relationships in efficiency between steps among genotypes may compromise the overall SE efficiency. Selecting clones of higher proliferation rates might be accompanied by a decrease, although statistically insignificant, in efficiencies of germination and acclimation (Table 6). Overall, the observed correlation among the SE steps was weak in general at both the family (Fig. 2) and clonal levels (Table 6), suggesting that genetic selection at one step will not negatively affect the efficiencies of other steps substantially. In *Picea glauca*, no correlation was found between the frequencies of SE initiation and maturation, or germination rates^27^, which parallels our finding in this study. Note that, however, the observed correlations in the present study might be spurious owing to small sample sizes. In terms of genetic selection efficiency, this is an important topic deserving further investigation by including more genotypes.

Currently, *Picea pungens* is mainly propagated by seeds either imported from North America or collected from seed production areas. For this species, seed production is unpredictable, varying from year to year, and, importantly, there is no guarantee that the favorable silvery blue color of needles in parents can be retained in their offspring via seed propagation, a drawback for ornamental species. Given the satisfactory results in the current study and the advantages of SE in deployment, incorporating the SE protocols described here into a genetic selection and propagation system for *Picea pungens* is promising. ET can be induced from mature embryos of seeds, with some being used for SE plant production for testing and others for cryopreservation; a tested superior genotype can immediately be recultured and mass propagated via SE. We used mature zygotic embryos as explants, which may reduce the efficiency of spruce SE in general^5,17,27^, is more useful operationally, as they can be extracted from seeds stored in seed banks or collected from seed production areas.

The present work has successfully established SE protocols for *Picea pungens*. Even though the SE protocols were appropriate overall, in order to incorporate the techniques into a commercial forestry program of *Picea pungens*, more studies are needed. The protocols described here might be suboptimal and can be improved in efficiency. In addition to further modifying the SE protocols, future research should concentrate on the cryopreservation of ET as well as subsequent topics, such as somaclonal variation, somatic embryo recovery, and seedling growth in the field. Another issue is high cost, which may be reduced by automating SE steps^28^*.* The high cost of SE plants can be a limitation for any commercial species, but for ornamental species, such as *Picea pungens*, a high price for high-quality trees can always be commanded. Overall, the results of this study are encouraging in terms of efficiency, and the protocols can be used as a foundation for further commercializing SE techniques of *Picea pungens.*
